# Ranking hospital performance based on individual indicators: can we increase reliability by creating composite indicators?

**DOI:** 10.1186/s12874-019-0769-x

**Published:** 2019-06-26

**Authors:** Peter C. Austin, Iris E. Ceyisakar, Ewout W. Steyerberg, Hester F. Lingsma, Perla J. Marang-van de Mheen

**Affiliations:** 1 ICES, G106, 2075 Bayview Avenue, Toronto, Ontario Canada; 2 Department of Public Health, Erasmus MC, Dr. Molewaterplein 40, 3015 GD Rotterdam, The Netherlands; 3 Department of Biomedical Data Sciences, Medical Decision Making, Leiden University Medical Centre, PO Box 9600, 2300 RC Leiden, The Netherlands

**Keywords:** Reliability, Rankability, Performance indicators, Hospital performance, Provider profiling

## Abstract

**Background:**

Report cards on the health care system increasingly report provider-specific performance on indicators that measure the quality of health care delivered. A natural reaction to the publishing of hospital-specific performance on a given indicator is to create ‘league tables’ that rank hospitals according to their performance. However, many indicators have been shown to have low to moderate rankability, meaning that they cannot be used to accurately rank hospitals. Our objective was to define conditions for improving the ability to rank hospitals by combining several binary indicators with low to moderate rankability.

**Methods:**

Monte Carlo simulations to examine the rankability of composite ordinal indicators created by pooling three binary indicators with low to moderate rankability. We considered scenarios in which the prevalences of the three binary indicators were 0.05, 0.10, and 0.25 and the within-hospital correlation between these indicators varied between − 0.25 and 0.90.

**Results:**

Creation of an ordinal indicator with high rankability was possible when the three component binary indicators were strongly correlated with one another (the within-hospital correlation in indicators was at least 0.5). When the binary indicators were independent or weakly correlated with one another (the within-hospital correlation in indicators was less than 0.5), the rankability of the composite ordinal indicator was often less than at least one of its binary components. The rankability of the composite indicator was most affected by the rankability of the most prevalent indicator and the magnitude of the within-hospital correlation between the indicators.

**Conclusions:**

Pooling highly-correlated binary indicators can result in a composite ordinal indicator with high rankability. Otherwise, the composite ordinal indicator may have lower rankability than some of its constituent components. It is recommended that binary indicators be combined to increase rankability only if they represent the same concept of quality of care.

## Background

There is an increasing interest in reporting on the quality of health care and comparing the quality of health care and outcomes of treatment between health care providers. Several American states have released hospital report cards comparing patient outcomes between hospitals for patients hospitalized with acute myocardial infarction or undergoing coronary artery bypass graft surgery [1–6]. Similar reports have been released in the Canadian province of Ontario and in Scotland [7–9].

An indicator is either an outcome (e.g., mortality, surgical site infection, or length of stay) or a process of care (e.g., discharge prescribing of evidence-based medications in specific patient populations) that is used to assess the quality of health care. A common practice is to report hospital-specific means of health care indicators (e.g., the proportion of patients who died in each hospital or mean length of stay). Crude (or unadjusted) or risk-adjusted estimates of hospital performance on specific indicators can be reported.

When hospital-specific performance on indicators are reported, a natural tendency is to create ‘league tables’, in which hospitals are ranked according to their performance on a given indicator [10]. Implicit in such comparisons is the assumption that the indicator permits hospitals to be ranked accurately according to their performance on the indicator. However, such rankings do not account for inherent variability in ranking due to natural variation in the indicator. In a study on the use of empirical Bayes methods to assess health care quality, van Houwelingen et al. appear to have coined the term ‘rankability’ to refer to the ability to accurately rank hospitals [11]. While rankability is defined formally in the next section, it can be interpreted as the proportion of the variation between providers (in terms of the indicator) that is due to true differences (as opposed to natural variation due to unexplained factors). Potential values for the rankability of an indicator range between zero and one, with higher values suggesting that the indicator can be used to accurately rank hospitals. Lingsma et al. suggested that an indicator with a rankability above 0.7 can be considered to have high rankability [12]. A similar concept is referred to as ‘statistical reliability’ by others [13]. This concept has been implemented for both diagnostic and process indicators [14], as well as for outcome indicators in different fields [15–18].

Some indicators have been shown to have high rankability. Using pregnancy rate as an indicator for assessing the quality of a set of large IVF clinics was found to have a rankability of 0.90 [12]. Surgical site infection (SSI) after colonic resection had a rankability of 0.78 after adjusting for patient case-mix [19]. However, other indicators have been shown to have poor to moderate rankability. SSI across several types of surgery combined had a rankability of 0.08 after adjusting for patient case mix [19]. The indicator denoting poor outcome following hospitalization for stroke was shown to have a rankability of 0.55 [20]. Van Dishoeck examined seven indicators used in Dutch hospitals and found that only one had high rankability (unintended reoperation after colorectal surgery – rankability of 0.71; other indicators had rankability ranging from 0 to 0.58) [21]. Lawson examined the rankability of SSI following colorectal surgery and found that the mean rankability was 0.65 for superficial SSI, 0.40 for deep/organ-space SSI, and 0.59 for any SSI [22]. Hofstede et al. examined the rankability of in-hospital mortality for a variety of conditions or procedures [23]. They found that rankability ranged from 0.01 for patients with osteoarthritis undergoing total hip arthroplasty/total knee arthroplasty to 0.71 following hospitalization for stroke.

High rankability is a desirable property for an indicator, as it means that the indicator permits accurate ranking of hospitals or providers. In the context of randomized controlled trials it has been shown that ordinal outcomes result in more reliable estimates of the treatment effect than binary outcomes [24–26]. A question when developing indicators for assessing quality of health care is whether several binary indicators reflecting outcomes of increasing severity, which individually have poor to moderate rankability, can be combined into an ordinal indicator to increase rankability.

The objective of the current study was to examine how the rankability of composite ordinal indicators compared to the rankabilities of the component binary indicators. The paper is structured as follows: In Section 2, we provide background and formally define rankability. In Section 3, we conduct a series of Monte Carlo simulations to examine the relationship between the rankability of a binary indicator and the intraclass correlation coefficient (ICC) of that indicator across hospitals (as a measure of the between-hospital variation). In Section 4, we conduct a series of Monte Carlo simulations to examine the relationship between the rankability of a composite ordinal indicator and the rankabilities of the individual binary indicators from which it was formed. Finally, in Section 5 we summarize our findings and place them in the context of the existing literature.

## Rankability and notation

Let Y denote a binary indicator that is used to assess the performance of a health care provider (e.g., hospital or physician). Throughout the manuscript, we will refer to the hospital as the provider, but the methods are equally applicable to other healthcare providers (e.g., physicians or health care administrative regions). *Y*_*ij*_ = 1 denote that the indicator was positive or present (e.g., the patient died or SSI occurred) for the *i*th patient at the *j*th hospital, while *Y*_*ij*_ = 0 denotes that the indicator was negative for this patient (e.g., the patient did not die or SSI did not occur). Let **X**_*ij*_ denote a vector of covariates measured on the *i*th patient at the *j*th hospital (e.g., age, sex, and comorbid conditions).

A random effects logistic regression model can be fit to model the variation in the indicator:

1 $$ \mathrm{logit}\left(\Pr \left({Y}_{ij}=1|{\mathbf{X}}_{ij}\right)\right)=\beta {\mathbf{X}}_{ij}+{\alpha}_j $$

where *α*_*j*_ denotes a hospital-specific random effect that is assumed to be normally distributed: *α*_*j*_~*N*(*α*_0_, *τ*^2^) (we assume that **X**_*ij*_ does not contain a constant or intercept term). The random effects model allows one to formally model between-hospital variation in the indicator after adjusting for baseline covariates. The ICC or the variance partition coefficient (VPC) can be calculated using the latent variable approach as $\mathrm{ICC}=\frac{\tau^2}{\tau^2+\frac{\pi^2}{3}}$, where τ^2^ is the variance of the hospital-specific random effects defined above and π is the mathematical constant [27, 28]. The ICC denotes the proportion of the variation in the indicator that is due to systematic between-hospital variation in the indicator. While there are multiple definitions of the ICC for use with clustered data [29], we used the above definition because it appears to be the most frequently used definition in the context of multilevel analysis.

Instead of fitting a random effects model to model variation in the indicator, one could replace the hospital-specific random effects by fixed hospital effects:

2 $$ \mathrm{logit}\left(\Pr \left({Y}_{ij}=1|{\mathbf{X}}_{ij}\right)\right)=\beta {\mathbf{X}}_{ij}+{\alpha}_2\mathrm{I}\left(j=2\right)+\cdots +{\alpha}_k\mathrm{I}\left(j=\mathrm{k}\right) $$

where there are k-1 indicator or dummy variables to represent the fixed effects of the k hospitals. Let *s*_*j*_ denote the standard error of the estimated hospital effect for the *j*th hospital. These standard errors denote the precision with which the hospital-specific fixed effects are estimated.

The rankability or reliability of the binary indicator is defined as $\rho =\frac{\tau^2}{\tau^2+\mathrm{median}\left({s}_j^2\right)}$, where *τ*^2^ and ${s}_j^2$ are as defined above [20]. The rankability relates the total variation from the random effects model to the uncertainty of the individual hospital effects from the fixed effects model. It can be interpreted as the proportion of the variation between hospitals that is not due to chance.

When considering an ordinal indicator with three or more levels, rankability can be defined similarly through the use of ordinal regression models. Model (1) is replaced by a random effects ordinal logistic regression model, while Model (2) is replaced by a fixed effects ordinal logistic regression model.

## Monte Carlo simulations to examine the relationship between ICC and rankability for a single binary indicator

We conducted a series of Monte Carlo simulations to examine the relationship between ICC and the rankability of a single binary indicator.

### Methods

Let X and Y denote a continuous risk score and a binary indicator, respectively. The following random effects model relates the continuous risk score to the presence of the binary indicator:

3 $$ \mathrm{logit}\left(\Pr \left({Y}_{ij}=1\right)\right)={\alpha}_{0j}+{\alpha}_1{X}_{ij} $$

The hospital-specific random effects follow a normal distribution: *α*_0*j*_~*N*(*α*_0_, *τ*^2^). The average intercept, *α*_0_, determines the overall prevalence of the binary indicator, while the slope, *α*_1_, determines the magnitude of the strength of the relationship between the risk score and the presence of the binary indicator. Fixing the standard deviation of the random effects distribution at $\tau =\pi \sqrt{\frac{\mathrm{ICC}}{3\left(1-\mathrm{ICC}\right)}}$ will result in a model with the desired value of the ICC.

We simulated data for 500 patients at each of 100 hospitals. For each of the 100 hospitals, we simulated a hospital-specific random intercept: *α*_0*j*_~*N*(*α*_0_, *τ*^2^). The value of *τ*^2^ was chosen to produce a desired ICC. For each subject, a risk score was simulated from a standard normal distribution: *x*_*ij*_~*N*(0, 1). Then, for each subject we computed the linear predictor using formula (3). We then simulated a binary outcome for the indicator from a Bernoulli distribution with subject-specific parameter Pr(*Y*_*ij*_ = 1). In practice, hospital volume varies across hospitals. We designed the simulations so that hospital volume was fixed across hospitals. This was done to remove any effect of varying hospital volume on rankability.

We allowed the following three factors to vary: (i) the ICC; (ii) the average intercept (*α*_0_); (iii) the fixed slope (*α*_1_). The ICC was allowed to take on 13 values from 0 to 0.24 in increments of 0.02. These values were selected as they range from no effect of clustering (ICC = 0) to a strong effect of clustering. The average intercept was allowed to take on four values: − 3, − 2, − 1.5, and − 1. The fixed slope was allowed to take on three values: − 0.25, 0, and 0.25. We used a full factorial design, and thus considered 156 different scenarios.

In each of the 156 different scenarios we simulated 100 datasets. In each of the 100 simulated datasets, we estimated the rankability of the binary indicator using the methods described in Section 2 (in each simulated dataset rankability was estimated using the estimated variance of the random effects, rather than the known true value). For a given scenario, we then computed the average rankability across the 100 simulated datasets for that scenario. The simulations were conducted using the R statistical programming language (version 3.5.1). The random effects logistic regression models were fit using frequentist methods using the glmer function from the lme4 package for R.

### Results of the Monte Carlo simulations

**Fig. 1:**
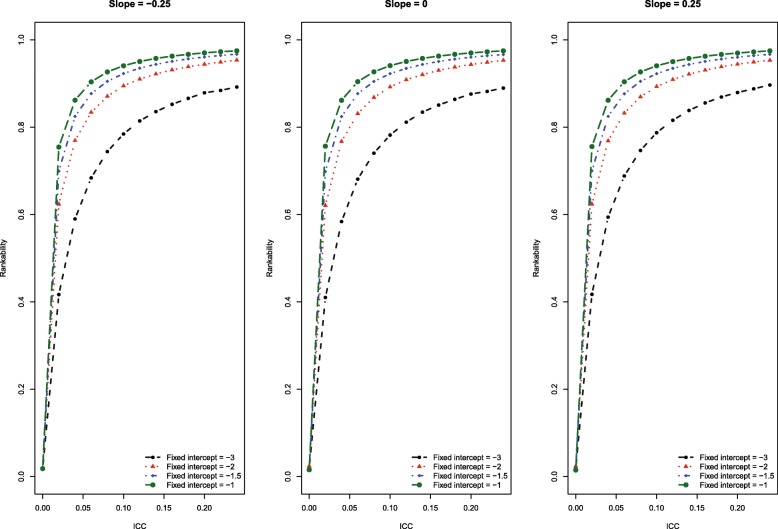
Effect of ICC on rankability

The results of the Monte Carlo simulations are summarized in Fig. 1. The figure consists of three panels, one for each of the three fixed slopes relating the risk score to the presence of the indicator. Each panel shows the relationship between ICC and rankability for the four scenarios defined by the four values for the average intercept. Several patterns warrant being highlighted. First, for a given value of the average intercept, rankability increased with increasing values of ICC. Second, for a given value of the ICC, rankability increased as the average intercept increased from − 3 to − 1. Third, for a given value of ICC and average intercept, rankability was negatively correlated with the fixed slope. Fourth, either the average intercept (i.e., the overall prevalence of the indicator) had to be moderate to large (− 2 to − 1) or the ICC had to be high for rankability to exceed the 0.7 (70%) threshold that was previously proposed to denote reasonable rankability [12].

## Monte Carlo simulations to examine reliability of composite indicators

We used an extensive series of Monte Carlo simulations to examine whether combining three binary indicators into an ordinal indicator resulted in an ordinal indicator with greater rankability compared to that of its binary components.

### Methods

We examined scenarios with three binary indicators: Y_1_, Y_2_, and Y_3_. The following three random effects models relate an underlying continuous risk factor to the presence of each of the three binary indicators:

4 $$ \left\{\begin{array}{l}\mathrm{logit}\left(\Pr \left({Y}_{1 ij}=1\right)\right)={\alpha}_{01j}+{\alpha}_{11}{X}_{ij}\\ {}\mathrm{logit}\left(\Pr \left({Y}_{2 ij}=1\right)\right)={\alpha}_{02j}+{\alpha}_{12}{X}_{ij}\\ {}\mathrm{logit}\left(\Pr \left({Y}_{3 ij}=1\right)\right)={\alpha}_{03j}+{\alpha}_{13}{X}_{ij}\end{array}\right. $$

As above, for a given random effects model, we assumed that the hospital-specific random effects followed a normal distribution: ${\alpha}_{0 kj}\sim N\left({\alpha}_{0k},{\tau}_{kk}^2\right)$, for *k* = 1, 2, 3. We assumed that the distribution of the triplet of hospital-specific random effects followed a multivariate normal distribution:

5 $$ \left(\begin{array}{l}{\alpha}_{01j}\\ {}{\alpha}_{02j}\\ {}{\alpha}_{03j}\end{array}\right)\sim \mathrm{MVN}\left(\left(\begin{array}{l}{\alpha}_{01}\\ {}{\alpha}_{02}\\ {}{\alpha}_{03}\end{array}\right),\left(\begin{array}{l}{\tau}_{11}^2\kern1em {\tau}_{12}\kern1em {\tau}_{13}\\ {}{\tau}_{21}\kern1em {\tau}_{22}^2\kern1em {\tau}_{23}\\ {}{\tau}_{31}\kern1em {\tau}_{32}\kern1em {\tau}_{33}^2\end{array}\right)\right) $$

We considered scenarios in which the prevalences of the three indicators across all hospitals were 0.05, 0.10, and 0.25 (Pr(*Y*_1*ij*_ = 1) = 0.05, Pr(*Y*_2*ij*_ = 1) = 0.10, and Pr(*Y*_3*ij*_ = 1) = 0.25) as this is typical the range of prevalences occurring frequently in practice. For instance, Hofstede et al. found that the median hospital-specific rate of in-hospital mortality amongst patients with colorectal carcinoma was 4.9%, while the median acute readmission rate for stroke patients was 6.1% [23]. They found that the median in-hospital mortality rate for patients with heart failure was 11.0%, while the acute readmission rate for colorectal carcinoma patients was 10.7%. Van Dishoeck et al. found that the median rate of having remaining cancer tissue after breast-saving lumpectomy was 10.5% [21]. Finally, long length of stay (LOS) has been defined as a LOS that is in the top 25% for patients with a given diagnosis or procedure [23]. This indicator would have an overall prevalence of 25% by construction.

Informed by the results of the first set of simulations, we fixed the three slopes relating the continuous risk score to the presence of the three binary indicators as follows: *α*_11_ =  − 0.25,  *α*_12_ = 0.50,  *α*_13_ = 1. We then used a bisection approach to determine appropriate values for *α*_0*j*_,  *j* = 1, 2, 3 such that the indicators had the desired prevalence. We then used a grid search to select values of ${\tau}_{jj}^2,\kern0.5em j=1,2,3$ to result in simulated data such that the simulated binary indicators had low (rankability < 0.5 [12]) to moderate (rankability from 0.5 to 0.7 [12]) rankability.

For a given scenario, we simulated 100 datasets, consisting of N patients at each of 100 hospitals (this is approximately equal to the number of hospitals in The Netherlands, where most of the authors are located, and thus may be typical of the number of hospitals in small countries). Within each simulated dataset we computed the rankability of the three binary indicators. We also created a five-level ordinal indicator created by combining the three binary indicators. Our five-level ordinal indicator was created so as to go from best (or least serious/severe) (a value of 1) to worst (or most serious/severe) (a value of 5). It was motivated by scenarios in which the three binary indicators denote outcomes of differing severities and that have different prevalences. In particular, the first indicator is the most severe or serious of the three indicators and also occurs the least frequently (e.g., death); the third indicator is the least severe or serious and also occurs the most frequently (e.g., long hospital length of stay); the second indicator is intermediary in terms of both severity/seriousness and prevalence (e.g., subsequent hospital readmission). A previous empirical study examined an ordinal composite indicator created by pooling these three binary indicators with these properties [23]. The ordinal indicator in our study was defined as:

6 $$ {Y}_{ij}=\left\{\begin{array}{l}5\ \mathrm{if}\ {Y}_{1 ij}=1\\ {}4\ \mathrm{if}\ {Y}_{2 ij}=1\ \mathrm{and}\ {Y}_{3 ij}=1\\ {}3\ \mathrm{if}\ {Y}_{2 ij}=1\ \mathrm{and}\ {Y}_{3 ij}=0\\ {}2\ \mathrm{if}\ {Y}_{3 ij}=1\ \mathrm{and}\ {Y}_{2 ij}=0\\ {}1\ \mathrm{otherwise}\end{array}\right. $$

Thus, a subject had the most severe/serious level of the composite ordinal indicator (5) if the most serious of the binary indicators (Y_1_) was present, regardless of whether or not any of the other two indicators had occurred. A subject had the least severe/serious level of the composite ordinal indicator (1) if none of the binary indicators was present. We computed the rankability of the ordinal indicator. The mean rankability of each of the three binary indicators and the one ordinal indicator was determined over 100 iterations for each scenario.

We allowed two factors to vary in the above simulations: (i) the number of subjects per hospital; (ii) the correlations between the hospital-specific random effects (cor(*α*_0*kj*_, *α*_0*lj*_),  *k* ≠ *l*). We considered two levels for the number of subjects per hospital: 500 and 1000. We considered eight values for the correlation between hospital-specific random effects: − 0.25, − 0.10, 0, 0.10, 0.25, 0.50, 0.75, and 0.90. Thus, we considered indicators that were uncorrelated, weakly correlated, moderately correlated, and strongly correlated and also allowed both positive and negative correlations, as found in practice [30]. For each of the 16 combinations of the above two factors we considered three different sets of rankability values for the three binary indicators. We thus considered 48 different scenarios. The simulations were conducted using the R statistical programming language (version 3.5.1). The random effects logistic regression models were fit using frequentist methods using the glmer function from the lme4 package for R. The ordinal logistic regression model was fit using the polr function from the MASS package, while the random effects ordinal logistic regression model was fit using the clmm function from the ordinal package for R.

### Results of the Monte Carlo simulations

The mean prevalence of the first, second, and third binary indicators across the 48 scenarios were 0.05, 0.10 and 0.25, respectively. The mean rankability of the first, second, and third binary indicators across the 48 scenarios were 0.36 (range 0.22 to 0.43), 0.46 (range 0.29 to 0.59), and 0.52 (range 0.33 to 0.71), respectively.

**Fig. 2:**
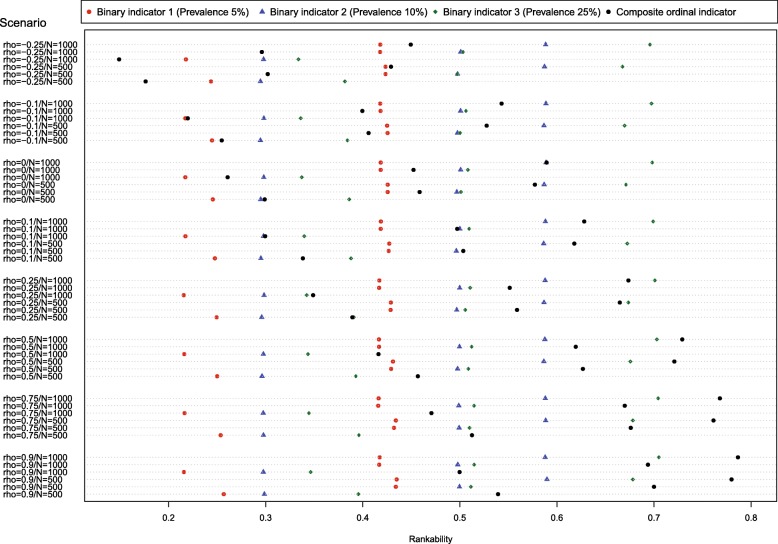
Rankability of binary and ordinal indicators

The results of the second set of Monte Carlo simulations are reported in Fig. 2. The results are reported using a dot chart. There is one row for each of the 48 scenarios (for each of the 16 combinations of number of subjects per hospital and correlation of the random effects, we considered three different sets of rankabilities for the binary indicators). On each line there are four dots, denoting the mean rankability of the three binary indicators and of the ordinal indicator. In 22 (46%) of the 48 scenarios, the composite ordinal indicator had greater rankability than did any of the three binary indicators. The likelihood that the composite ordinal indicator had greater rankability than that of the three binary indicators increased as the correlation of the hospital-specific effects increased. When the correlation was negative or equal to zero, then the composite ordinal indicator never had greater rankability than that of each of the three binary indicators. When the correlation was equal to 0.10, then the composite ordinal indicator had greater rankability than that of the three binary indicators in 17% of the scenarios. When the correlation was equal to 0.25, then the composite ordinal indicator had greater rankability than that of the three binary indicators in 50% of the scenarios. When the correlation was greater than or equal to 0.50, then the composite ordinal indicator had greater rankability than that of the three binary indicators in 100% of the scenarios. In 26 (54%) of the 48 scenarios, the composite ordinal indicator had lower rankability than that of the binary indicator with the greatest rankability. Increasing hospital volume from 500 to 1000 patients did not have a discernible effect on the likelihood that the composite ordinal indicator had greater rankability than that of the three binary indicators. A high rankability of the composite indicator was only observed in simulations in which the three binary indicators had moderate rankability and were strongly correlated with one another. However, not all scenarios with the two latter characteristics yielded a composite indicator with a high rankability (Fig. 2).

We used linear regression estimated ordinary least squares to regress the rankability of the ordinal indicator on the following variables: the rankability of the three binary indicators, the correlation between the hospital-specific random effects, and the number of subjects per hospital. Number of subjects per hospital was treated as a categorical variable with two levels, while the remaining covariates were treated as continuous quantitative covariates. The estimated regression coefficients are reported in Table 1. The R^2^ statistic for the fitted model was 0.97 (as was the adjusted R^2^ statistic). Only two of the variables had an independent effect on the rankability of the composite ordinal indicator: the rankability of the indicator with prevalence 0.25 and the correlation between the hospital-specific random effects. The latter result supports our previous results in Fig. 2 that combining highly-correlated binary indicators can result in a composite ordinal indicator with rankability that exceeds that of its binary components. We repeated the regression analysis, restricting the analysis to those scenarios in which the correlation between hospital-specific random effects was less than or equal to 0.5, and obtained similar results.

**Table 1:** Regression analysis on simulation results

Variable	Estimate	Standard error	*P*-value
Intercept	−0.057	0.026	0.0341
Rankability of indicator 1 (prevalence = 5%)	0.382	0.281	0.1813
Rankability of indicator 2 (prevalence = 10%)	0.074	0.377	0.8455
Rankability of indicator 3 (prevalence = 25%)	0.603	0.188	0.0025
1000 patients per hospital (vs. 500 patients)	−0.001	0.011	0.9290
Correlation of random effects	0.293	0.011	< 0.0001

The use of 100 replications in each of the 48 scenarios in the Monte Carlo simulations allowed us to estimate rankability with relatively good precision. For each scenario and for each of the indicators we computed the standard deviation of the rankability across the 100 replications for that scenario. The mean standard deviation of the rankability of the first binary indicator was 0.067 across the 48 scenarios (ranging from 0.062 to 0.074). The mean standard deviation of the rankability of the second binary indicator was 0.058 across the 48 scenarios (ranging from 0.046 to 0.069). The mean standard deviation of the rankability of the third binary indicator was 0.056 across the 48 scenarios (ranging from 0.037 to 0.072). The mean standard deviation of the rankability of the composite ordinal indicator was 0.057 across the 48 scenarios (ranging from 0.032 to 0.078).

**Fig. 3:**
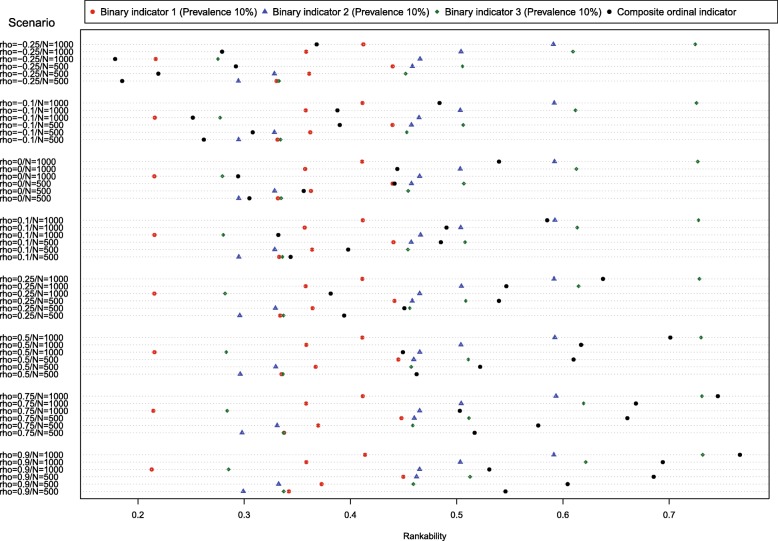
Rankability of binary and ordinal indicators (equal prevalences)

We conducted an additional set of simulations that were a modification of those reported above. In this additional set of simulations, the prevalence of all three indicators was set to 10% (instead of 5% vs. 10% vs. 25%). Results for these simulations are reported in Fig. 3. In 18 (38%) of the 48 scenarios, the composite ordinal indicator had greater rankability than did any of the three binary indicators. The likelihood that the composite ordinal indicator had greater rankability than that of the three binary indicators increased as the correlation of the hospital-specific effects increased. When the correlation was negative or equal to zero, then the composite ordinal indicator never had greater rankability than that of each of the three binary indicators. When the correlation was equal to 0.10, then the composite ordinal indicator had greater rankability than that of the three binary indicators in 17% of the scenarios. When the correlation was equal to 0.25, then the composite ordinal indicator had greater rankability than that of the three binary indicators in 33% of the scenarios. When the correlation was equal to 0.50, then the composite ordinal indicator had greater rankability than that of the three binary indicators in 50% of the scenarios. When the correlation was greater than or equal to 0.75, then the composite ordinal indicator had greater rankability than that of the three binary indicators in 100% of the scenarios. In 30 (63%) of the 48 scenarios, the composite ordinal indicator had lower rankability than that of the binary indicator with the greatest rankability.

## Discussion

We conducted a series of simulations to examine whether combining three binary indicators reflecting outcomes with increasing severity, which individually had low or moderate rankability, could produce an ordinal indicator with high rankability. We found that this was feasible when the three binary indicators had at least moderate rankability and were strongly correlated with one another. When the binary indicators were independent or weakly correlated with one another, the rankability of the composite ordinal indicator was often less than that of at least one of its binary components.

There is an increasing interest in many countries and jurisdictions in reporting on the quality and outcomes of health care delivery. Public reporting of hospital-specific performance on indicators of health care quality can lead to the production of ‘league tables’, in which hospitals are ranked according to their performance. The rankability of an indicator denotes its ability to allow for the accurate ranking of hospitals. As noted in the Introduction, many indicators have been shown to have poor to moderate rankability.

Our focus was on pooling binary indicators reflecting outcomes of increasing severity to create a composite ordinal indicator that described a gradient from lowest (least severe/serious) to highest (most severe/serious). We did not consider other methods of creating composite indicators such as summing up the number of positive binary indicators. Such an approach would not necessarily preserve the ordering of severity present in the individual indicators. For instance given three indicators of differing severity (e.g., death, hospital readmission, and long length of hospital stay), then a subject who died (and who was not readmitted and who had a short length of hospital stay) and a subject who had a long hospital stay (but who did not die and who was not readmitted) would both have one positive indicator. However, they would have very different severity of the underlying binary indicators. Our composite ordinal indicator reflects this ordering of severity/seriousness, while counting the number of positive indicators would not.

Our research has shown that rankability is increased when individual indicators are combined with other indicators with which they are highly correlated. Individual indicators underlying the same concepts of (quality) of care can thereby be combined to produce a more reliable ranking with the added advantage of showing a more complete picture of quality of care. On the other hand, indicators that are not correlated might represent other important quality domains. These should not be ignored, although their limited rankability should be taken into account in the interpretation of potential differences between hospitals.

Our results confirm that rankability is affected by the variation of the hospital-specific random effects, in other words the magnitude of the between-hospital differences, and by the overall prevalence of the outcome, influencing the reliability of the hospital-specific random effects. These terms are included in the definition of rankability. Further, we found that the one of the two factors with the strongest effect on the rankability of an ordinal outcome is the rankability of the most prevalent binary outcome. This is intuitive since the indicator with the highest prevalence contributes the most information to the ordinal outcome. Finally, our most important finding is that ordinal outcomes only increase rankability when the component binary indicators are strongly correlated (typically, the within-hospital correlation needed to be at least 0.5). This explains why a previous study found no increase in rankability when combining mortality, readmission and length of stay. These binary indicators were negatively correlated, partly by definition (e.g. high mortality will mean less readmissions), partly because they represent different aspects of quality of care [23]. The finding that combining binary outcomes that are negatively correlated, uncorrelated or only weakly correlated, into an ordinal outcome decreases rankability is a result of violation of the proportional odds assumption. The proportional odds model assumes that the effect of the parameter of interest, in this case the hospital-specific random effects, on the outcome is comparable across the cut-offs of the ordinal scale. If the binary indicators are not correlated this assumption is not satisfied. For example, when a specific hospital has a low mortality rate (meaning a negative random effect estimate on one cut-off) but high readmission rate (positive random effect estimate on other cut-off) these random effect estimates average out. This reduces the variation of the hospital-specific random effects, resulting in lower rankability. Thus, to obtain a composite ordinal indicator with high rankability, the proportional odds assumption must be met to some extent.

Combining binary indicators to form a composite ordinal indicator presents several issues that must be addressed. First, one must identify binary indicators whose combination would be meaningful for profiling health care provider performance. Patients may not be interested in one indicator at a given time (e.g., whether a readmission occurs), but may want to know the likelihood that success is achieved on a range of indicators (e.g., no readmission and normal length of stay), also called a textbook-outcome [23, 31]. Combining indicators is also important for record review by professionals if they want to improve quality, where the improvement may involve a different intervention for patients with a normal length of stay and a readmission (as they may be discharged too early) than for patients with a readmission after a long length of stay (which may reflect complex patients). Secondly, ideally, one must identify binary indicators with a strong within-hospital correlation (i.e., hospitals that have higher performance on one indicator also have higher performance on the other indicators), which is often not the case in practice [30]. Third, in order for a composite indicator to provide information on which a hospital can take action, it would be reasonable to combine indicators that address aspects of health care quality for the same set of patients (e.g., that pertain to the same surgical procedure or to the treatment of the same set of patients). Identifying indicators that satisfy these requirements may be challenging in some settings.

## Conclusion

Pooling highly-correlated binary indicators can result in a composite ordinal indicator with high rankability. However, when binary indicators have low to moderate within-hospital correlation, the composite ordinal indicator may have lower rankability than some of its constituent components. It is recommended that related binary indicators be combined in order to increase rankability, which reflects that they represent the same concept of quality of care.

## Data Availability

The datasets used and/or analysed during the current study are available from the corresponding author on reasonable request.

## Abbreviations

ICC: Intraclass correlation coefficient
IVF: In vitro fertilization
LOS: Length of stay
SSI: Surgical site infection
VPC: Variance partition coefficient
